# Adjuvant Anti-PD-1 Immunotherapy versus Conventional Therapy for Stage III Melanoma: A Real-World Retrospective Cohort Study

**DOI:** 10.3390/ph16010041

**Published:** 2022-12-28

**Authors:** Tong Li, Yu Xu, Wei Sun, Wangjun Yan, Chunmeng Wang, Tu Hu, Xiaowei Zhang, Zhiguo Luo, Xin Liu, Yong Chen

**Affiliations:** 1 Department of Pancreatic Surgery, Fudan University Shanghai Cancer Center, Shanghai 200032, China; 2 Department of Oncology, Shanghai Medical College, Fudan University, Shanghai 200032, China; 3 Shanghai Pancreatic Cancer Institute, Shanghai 200032, China; 4 Pancreatic Cancer Institute, Fudan University, Shanghai 200032, China; 5 Department of Musculoskeletal Oncology, Fudan University Shanghai Cancer Center, Shanghai 200032, China; 6 Department of Internal Medicine of Oncology, Fudan University Shanghai Cancer Center, Shanghai 200032, China; 7 Department of Gastrointestinal Oncology, Shanghai Medical College, Fudan University, Shanghai 200032, China; 8 Department of Head and Neck and Neuroendocrine Oncology, Shanghai Medical College, Fudan University, Shanghai 200032, China

**Keywords:** melanoma, programmed cell death 1, interferon, recurrence-free survival, distant metastasis-free survival, overall survival

## Abstract

The use of adjuvant therapy has provided survival benefits in patients with advanced melanoma. This study aimed to explore the recurrence and prognosis of the PD-1 inhibitor, conventional interferon (IFN), or observation (OBS) on resected stage III acral and cutaneous melanoma patients through a retrospective analysis. Patients with resected stage III melanoma at Fudan University Shanghai Cancer Center from 2017 to 2021 were enrolled with all of their clinicopathologic characteristics collected. They were divided into three groups: PD-1 inhibitor, IFN, and OBS. Survival analyses were performed to indicate the significance of different adjuvant therapies. A total of 199 patients were enrolled (PD-1 n = 126; IFN n = 31; and OBS n = 42), with their median follow-up times being 21 months, 24 months, and 49 months, respectively. The PD-1 inhibitor significantly improved relapse-free survival (*p* = 0.027) and overall survival (*p* = 0.033) compared with conventional treatment (IFN+OBS). The superiority of the PD-1 inhibitor was witnessed in stage IIIC/D (*p* = 0.000) acral (*p* = 0.05) melanoma patients with ulceration (*p* = 0.011) or lymph node macrometastasis (*p* = 0.010). The PD-1 inhibitor significantly reduced local recurrence and systemic metastasis compared with conventional therapy (*p* = 0.002). In conclusion, adjuvant anti-PD-1 immunotherapy can achieve better survival outcomes in acral and cutaneous melanoma patients compared with conventional treatment, without considering adverse events. More clinical benefits were seen in later-stage acral melanoma patients with ulceration or lymph node macrometastasis.

## 1. Introduction

Melanoma is highly invasive and is the most lethal cutaneous malignancy [1]. Primary prevention and early detection remain the fundamental method to improve the prognosis and survival of melanoma. Treatment decision making has become particularly difficult for advanced melanomas (stages III/IV) that have spread beyond the original tumor. The survival of patients in stages IIIA, B, C, and D remains 88%, 77%, 60%, and 24%, respectively, regardless of radical interventions [2], denoting the importance of adjuvant therapy, which could reduce the risk of recurrence and steer toward a better prognosis.

Recently, research on adjuvant therapy for melanoma has achieved substantial progress, with translation from trials to clinical practice still proceeding. Interferon (IFN) is the first approved adjuvant therapy for melanoma in Europe and has been conventionally utilized for decades, with different schedules being investigated (e.g., dosage regimen, with or without pegylation or induction), while yielding limited clinical benefits, especially for those with ulcerations [3,4,5]. Due to the intrinsic immunosuppressive features of melanoma, immune checkpoint inhibitors (ICIs) were adopted to help break the tolerance for melanoma [6]. Thereinto, programmed cell death 1 (PD-1) inhibitors (e.g., pembrolizumab, nivolumab, and toripalimab) could block the interplay between PD-1 and PD ligand 1/2 (PD-L1/2) and have been shown to provide remarkable improvement in relapse-free survival of eligible melanoma patients and thus have been recommended as a standard treatment instead of IFN [7,8,9].

Acral melanoma is the most common subtype of malignant melanoma in non-Caucasian populations including Asians [10,11], which has been closely associated with more advanced disease stages at presentation, deeper Breslow depth, and higher rates of ulceration and lymph node metastasis, as well as even poorer prognosis [12,13]. However, compared with ample clinical studies on cutaneous melanoma abroad, relatively large-scale, randomized controlled clinical studies of PD-1 monoclonal antibodies on acral melanoma are still limited. The data insufficiency on the effect of adjuvant PD-1 inhibitors for acral melanoma in Asia, as well as its comparison with that of other subtypes of melanoma, spurs us to investigate it. Therefore, in this study, we aim to explore the recurrence and prognosis of resected stage III acral and cutaneous melanoma patients treated by PD-1 inhibitor, conventional interferon (IFN), or simple observation (OBS) and identify a subgroup of patients who might have benefited in one of the most prominent Chinese cancer centers.

## 2. Results

### 2.1. Patient Characteristics

A total of 199 stage III melanoma patients were eligible for analysis, including 126 patients in the PD-1 inhibitor group, 31 patients in the IFN group, and 42 patients in the observation group. Overall, the demographics and clinicopathologic features of the patients in the three groups are presented in Table 1. All patients started receiving adjuvant therapy within one month after the surgery. The median follow-up times of each group were 21 months (PD-1 group), 24 months (IFN-α2b group), and 49 months (observation group).

### 2.2. Survival Data

#### 2.2.1. Relapse-Free Survival

**Figure 1:**
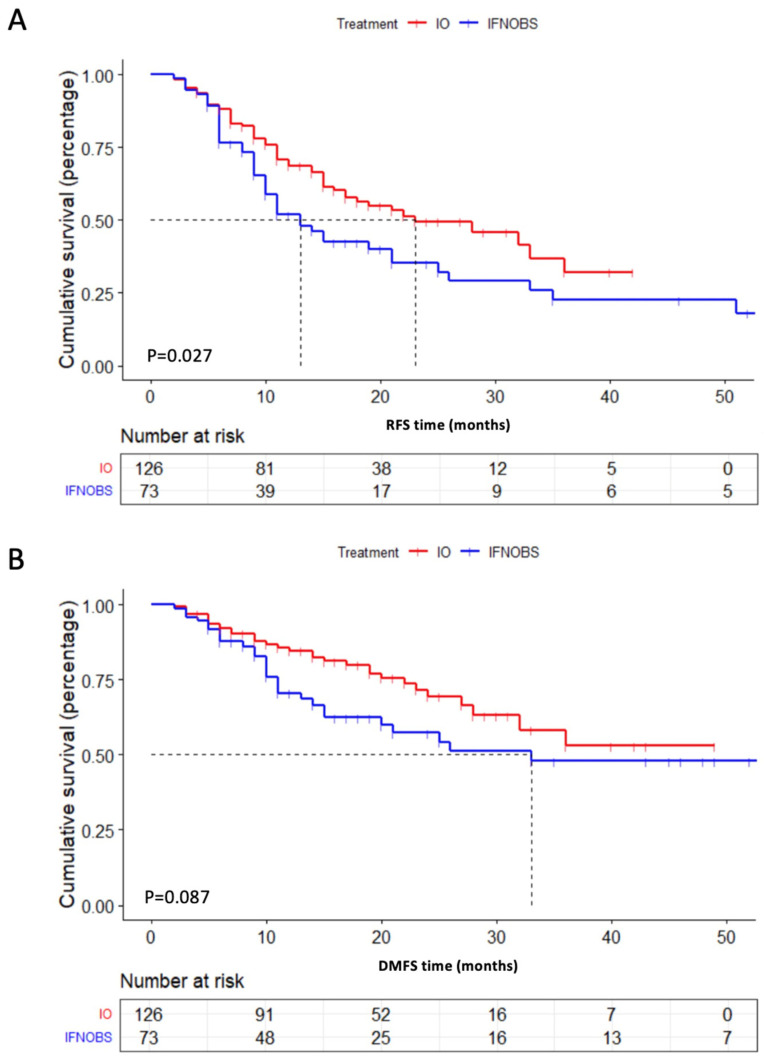
Kaplan–Meier curves of RFS (**A**) and DMFS (**B**) for all enrolled acral and cutaneous patients stratified by adjuvant PD-1 inhibitor treatment versus conventional treatment (IFN+OBS).

Recurrence was detected in 54 (42.8%) and 46 patients (63.0%) in the PD-1 inhibitor treatment group and conventional treatment (IFN+OBS) group, respectively, (*p* = 0.002). In addition, the treatment of PD-1 significantly reduced regional recurrence (22.2% vs. 26.0%) and systemic metastasis (20.6% vs. 37.0%) compared with conventional therapy (IFN+OBS) (*p* = 0.002) (Figure S1). At the time of this report, the median relapse-free survival (RFS) was 23 months in the PD-1 group, 15 months in the IFN group, and 11 months in the OBS group (Table S1). The 1-year RFS rates for the PD-1 group, IFN group, and OBS group were 70.0%, 59.2%, and 45.3%, while the 2-year RFS rates were 49.4%, 35.1%, and 37.4%, respectively. The treatment of PD-1 inhibitor caused a significantly longer RFS than the conventional therapy groups (OBS+IFN) (23 months vs. 13 months, *p* = 0.027) (Figure 1A) as well as simple observation (23 months vs. 11 months, *p* = 0.036) (Table S1).

**Figure 2:**
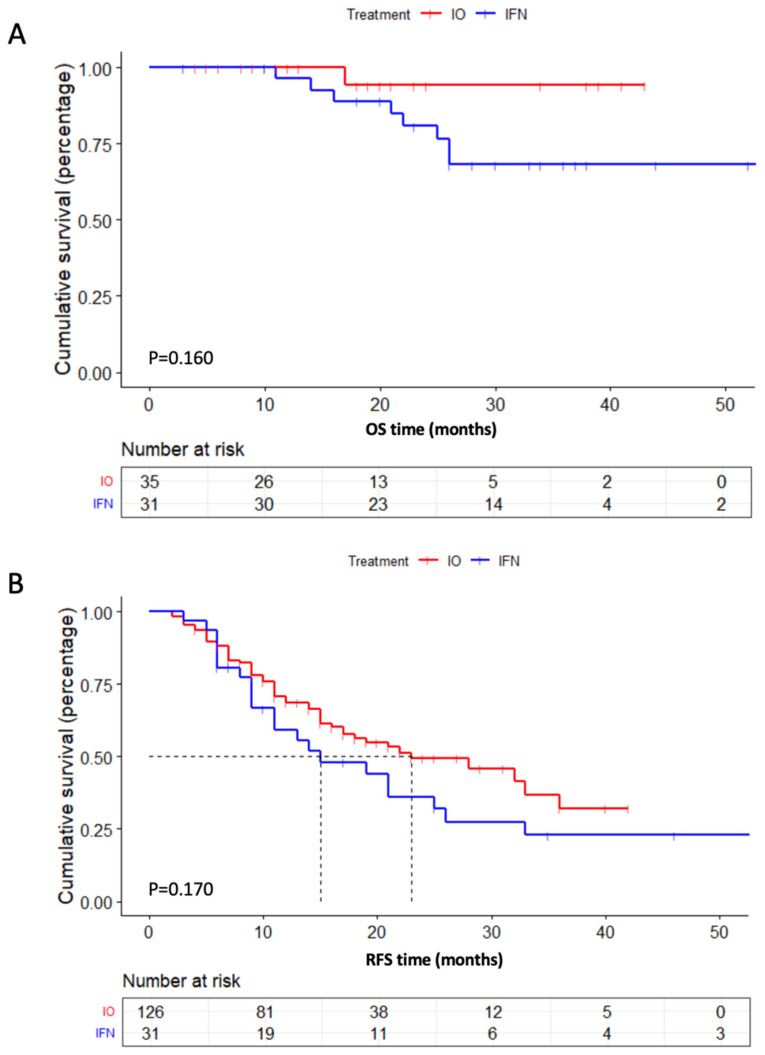
Kaplan–Meier curves of OS (**A**) and RFS (**B**) for all enrolled acral and cutaneous patients stratified by adjuvant PD-1 inhibitor treatment versus IFN treatment.

**Figure 3:**
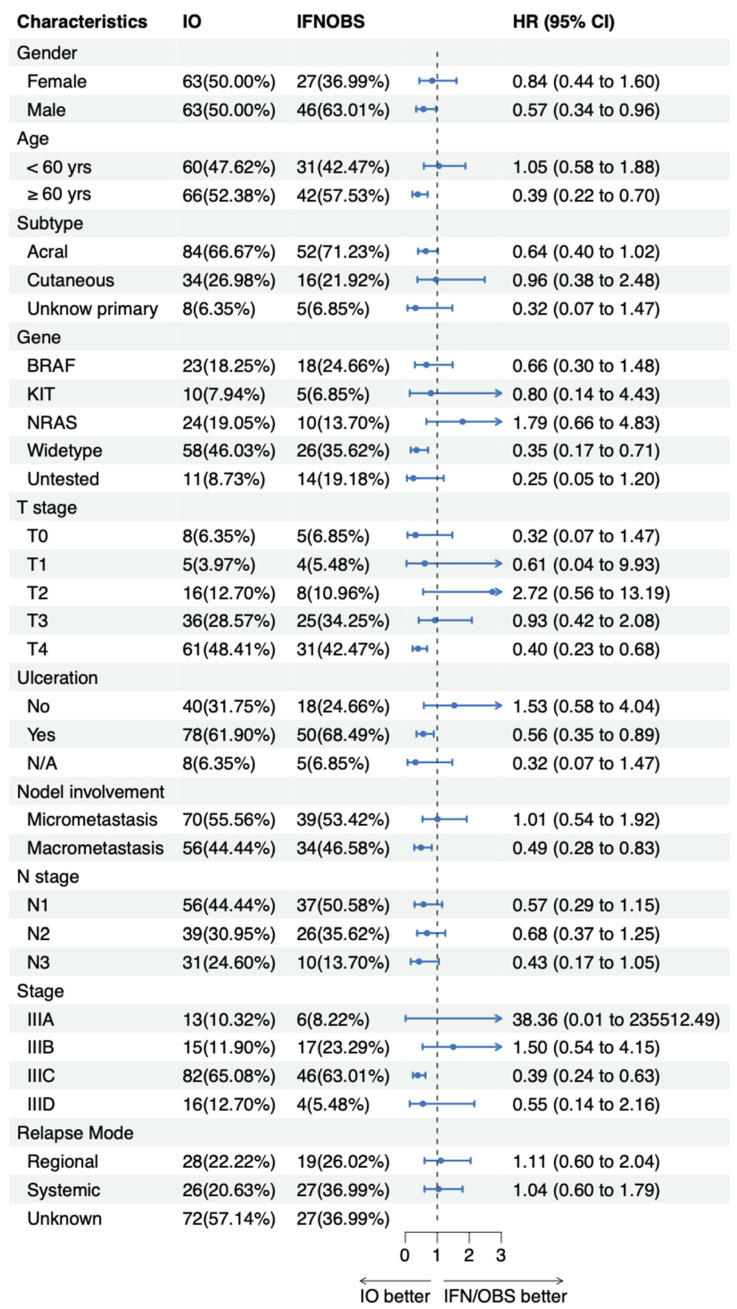
Forest plot of RFS according to subgroups. The hazard ratio of each subgroup is presented as green squares, while the 95% confidence intervals are presented as black lines.

No notable difference was observed in the RFS between the PD-1 inhibitor group and the IFN group (23 months vs. 15 months, *p* = 0.170) (Table S1). The overall survival (OS) was significantly longer for patients treated with PD-1 inhibitors than for those receiving IFN (*p* = 0.160) (Figure 2A). The RFS was 23 months (95% CI: 17-NA) for the PD-1 inhibitor group and 15 months (95% CI: 11–33) for the IFN group (*p* = 0.170) (Figure 2B). The among-group differences in RFS were consistent with observations across subgroups based on baseline characteristics shown in the forest plot (Figure 3). The data show that most subgroups could benefit from adjuvant PD-1 inhibitors versus conventional treatment groups (IFN+OBS).

#### 2.2.2. Distant Metastasis-Free Survival

At the time of this report, the median distant metastasis-free survival (DMFS) was not reached in the PD-1 inhibitor group, and it was 72 months in the IFN group and 20 months in the observation group (Table S2). The 1-year DMFS rates for the PD-1 group, IFN group, and OBS group were 84.7%, 85.3%, and 58.6%, while the 2-year DMFS rates were 69.4%, 68.5%, and 48.4%, respectively. The treatment of PD-1 inhibitor caused significantly longer DMFS than simple observation (NR vs. 20 months, *p* = 0.014). No notable difference was observed in the DMFS either between the PD-1 group and the IFN group (NR vs. 72 months, *p* = 0.106) or between the PD-1 group and the conventional groups (*p* = 0.087; Figure 1B).

#### 2.2.3. OS

**Figure 4:**
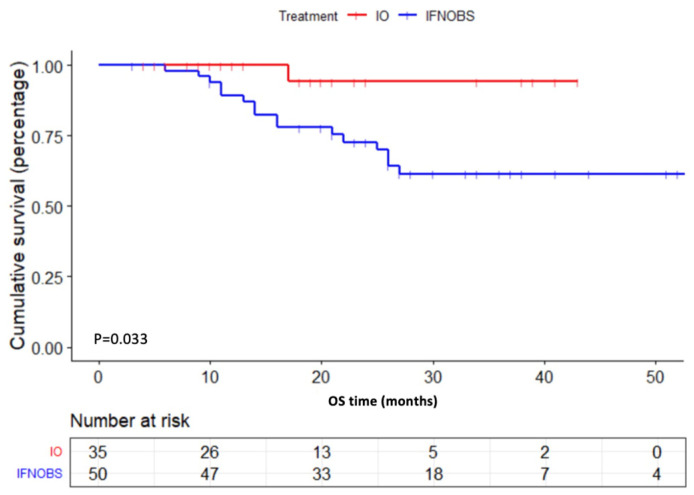
OS for all enrolled acral and cutaneous melanoma patients stratified by adjuvant PD-1 inhibitor treatment versus conventional treatment (IFN+OBS) (*p* = 0.033).

At the time of this report, the median OS of all groups was not reached. The treatment of PD-1 inhibitor caused significantly longer OS when compared with patients in the conventional therapy groups (OBS+IFN) (*p* = 0.033) (Figure 4) as well as the IFN group (*p* = 0.019) (Figure S3).

### 2.3. Subgroup Analysis

**Figure 5:**
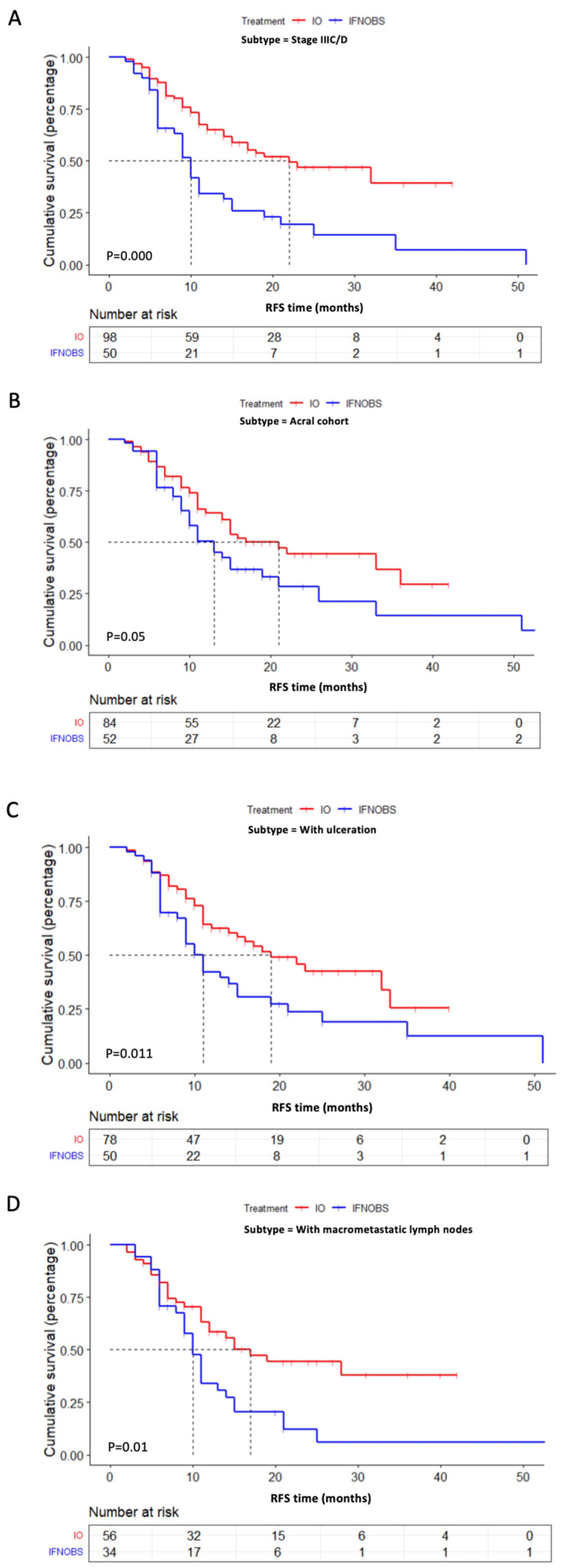
Kaplan–Meier curves of RFS for subgroups of acral and cutaneous patients stratified by adjuvant PD-1 inhibitor treatment versus conventional treatment (IFN+OBS). (**A**) RFS for all stage IIIC and IIID melanoma patients (*p* = 0.000). (**B**) RFS for all acral melanoma patients (*p* = 0.05). (**C**) RFS for all enrolled patients with ulceration (*p* = 0.011). (**D**) RFS for all melanoma patients with macrometastatic lymph nodes (*p* = 0.01).

For the patients with IIIC or IIID melanoma, the median RFS in the PD-1 group was significantly longer than that of the conventional IFN+OBS groups (*p* < 0.001) (Figure 5A) as well as the IFN group (*p* = 0.003) (Figure S4). Among the 136 stage III patients with acral melanoma, the treatment of PD-1 inhibitor was still longer than the IFN+OBS groups (*p* = 0.05) (Figure 5B), while no significance was observed between the PD-1 and the IFN groups (*p* = 0.075) (Figure S5). For 128 patients with ulceration, the treatment of PD-1 inhibitor significantly prolonged patients’ RFS when compared with patients in the IFN+OBS groups (*p* = 0.011) (Figure 5C), whereas no significance was found when compared only with the IFN group (*p* = 0.056) (Figure S6). For 90 patients with macrometastasis in the lymph nodes, PD-1 inhibitors significantly extended patients’ RFS more than that in the IFN+OBS groups (*p* = 0.01) (Figure 5D), while similar RFS results were found between the PD-1 and IFN groups (*p* = 0.075) (Figure S7).

## 3. Materials and Methods

### 3.1. Patients

Patients with resected melanoma at stage III who received an adjuvant PD-1 inhibitor, IFN-α2b monotherapy, or simple observation at Fudan University Shanghai Cancer Center (FUSCC, Shanghai, China) between January 2017 and December 2021 were retrospectively enrolled in this study. All histological specimens were evaluated by at least two experienced pathologists. Patients younger than 18 years old, pathologically diagnosed with mucosal melanoma, presented with baseline distant metastasis, or with a follow-up of less than six months were excluded. Finally, 199 patients were identified. This retrospective single-center study was conducted following the principles of the Declaration of Helsinki and was approved by the Institutional Review Board and Ethics Committee of the FUSCC. Because unidentified health data of patients were used, informed consent was waived.

### 3.2. Study Design

According to the same protocols in terms of resection, the patients were divided into three groups according to the treatment regimens: PD-1, IFN, and OBS. The patients in the PD-1 group received an intravenous infusion of pembrolizumab at a dose of 100 mg (weighing < 60 kg) or 200 mg (weighing ≥ 60 kg) or 240 mg of toripalimab every 3 weeks. The patients in the IFN-α2b group received a median or low dose of IFN-α at 3MIU to 6 MIU three times a week. Patients received adjuvant therapy for at most one year, or until disease recurrence or the emergence of unacceptable toxic events. All the patients received the same protocols in terms of the timing of surveillance and check-ups. The adverse events of the treatment were assessed according to the National Cancer Institute Common Terminology Criteria for Adverse Events (CTCAE v4.0) classification.

### 3.3. Data Retrieval and Follow-Up

The following clinicopathological information was retrieved including gender, age, pathological subtype, gene mutation, T stage, ulceration, nodal involvement, N stage, stage III subgroup, and relapse mode (initial). The pathological tumor (pT) stage, pathologic nodal (pN) stage, and pathological stage were defined according to the 8th edition of the *American Joint Committee on Cancer* (AJCC) cancer staging manual [2]. Patients were monitored via routine physical check-ups, ultrasound, CT scans, and/or MRI every 3 months for the first 2 years, every 6 months for 3–5 years, and then annually. Patient follow-up was performed via telephone until death or 30 May 2022. RFS was defined as the time interval from radical surgery to initial recurrence or death from any cause. DMFS was defined as the time interval from radical surgery to initial distant metastases or death from any cause. OS was defined as the time interval from radical surgery to death from any cause. Patients alive or lost to follow-up were censored.

### 3.4. Statistical Analyses

The RFS, DMFS, and OS were estimated by the Kaplan–Meier method, with the significance evaluated by the log-rank test. All statistical analyses were performed using IBM SPSS Statistics software (v 21.0). A two-tailed *p*-value of less than 0.05 was considered statistically significant.

## 4. Discussion

In this retrospective study involving Chinese patients with resected stage III melanoma, PD-1 inhibitors not only significantly prolonged the RFS but also contributed to prolonging DMFS compared with conventional therapy (IFN+OBS). PD-1 inhibitor therapy can bring especially more survival benefits for acral melanoma patients with ulceration, macrometastasis in lymph nodes, or regional recurrence. In addition, more survival benefits were found in patients with later stages (IIIC/D), especially in the control of distant metastasis than traditional treatment.

Tasuku Honjo et al. first discovered the PD-1 checkpoint in 1992 [14], which led to the development of nivolumab and pembrolizumab and their use in the adjuvant therapy of melanoma. The CHECKMATE 238 trial demonstrated that nivolumab can significantly extend RFS and DMFS even compared with the then-celebrated CTLA-4 inhibitor ipilimumab in stage IIIB/C or IV disease-free melanoma patients [15,16]. A subsequent analysis of patients with in-transit metastases (ITM) demonstrated significantly improved RFS and DMFS (HR 0.79; 95% CI 0.63–0.99) in the nivolumab group. The KEYNOTE-054 trial compared pembrolizumab and placebo in resected stage IIIA/B/C melanoma patients and showed remarkable improvement in the pembrolizumab group in both RFS (HR 0.57; CI 98.4%, 0.43–0.74; *p* < 0.0001) and DMFS (HR 0.60; 95% CI 0.49–0.73, *p* < 0.0001), irrespective of the PD-L1 status [17]. In addition, using pembrolizumab significantly reduced the onset of distant metastasis as the first event of relapse and reduced the percentage of loco-regional recurrence alone [18]. Moreover, the COMBI-AD trial showed that D+T (Dabrafenib+Trametinib) adjuvant therapy is also currently recommended for BRAF-mutant stage III melanoma patients. Nevertheless, the common limitation of these studies at present is that most of the patients recruited were with cutaneous melanoma, with only 29 cases (8.63%) of the acral subtype in the CHECKMATE 238 trial [19]. Acral melanoma is the most common subtype in the Asian population including Chinese and Japanese [20].

Moreover, the efficacy of PD-1 inhibitors in acral melanoma and the Chinese population remains controversial. Whether in the KEYNOTE151 or Polaris-01 studies, the response rate of pembrolizumab and toripalimab in advanced acral melanoma was only around 15%, which was far less than that of the cutaneous melanoma reported in trials conducted in the Western world [21,22]. At the same time, the ethnic discrepancy in clinical benefits exists, with studies showing that the immunotherapy’s efficacy in Asians, Hispanics, and Africans is worse than that in Caucasians [23]. Therefore, the efficacy of PD-1 inhibitors in the adjuvant treatment of acral melanoma still lacks an evidence base. In our study, the acral subtype accounted for 68.3% (136/199) of all cases. The estimated 1- and 2-year RFS rates in the PD-1 group were 68.6% and 49.4%, respectively, which was slightly worse than that of the KEYNOTE-054 study [24]. The results of the Japanese subgroup analysis from the CheckMate 238 study also showed poor efficacy in acral melanoma patients [25]. In brief, all the results above are consistent with the treatment outcomes of advanced cases, suggesting the poor prognostic immune features of the tumor microenvironment as well as compromised efficacy of adjuvant anti-PD-1 therapy in melanoma (especially the acral subtype).

Pembrolizumab shows great efficacy in patients with PD-L1-positive solid tumors. As indicated by the KEYNOTE053/S1404 trial, the pembrolizumab group can achieve better RFS (HR 0.74; 99.62% CI 0.57–0.96, *p* < 0.001) compared with the conventional IFN or ipilimumab group [26,27], which could be contributed by the high proportion (82%) of patients with PD-L1-positive tumors in baseline biopsies. However, the PD-L1 low/negative expression feature of the Chinese cohort indicates the inapplicable situation in the Chinese population. Lian et al. [28] compared the adjuvant regimens of toripalimab with IFN in resected mucosal melanoma patients. It showed that only 51.0% of the Chinese patients enrolled were PD-L1-positive, far fewer than Caucasians. They also indicated that despite the similar survival outcomes between toripalimab and high-dose interferon alpha-2b (HDI), toripalimab showed a more favorable safety profile than HDI, with a significantly lower incidence of treatment-emergent adverse events with a grade ≥3 (27.4% vs. 87.5%). Therefore, compared with HDI, PD-1 inhibitor may be more qualified as the treatment option for patients with resected melanoma, at least from a safety perspective.

IFN-α2b has long been used in standardized adjuvant therapy for stage IIB/III melanoma patients, which has been proven by the E1684, E1690, E1694, and E2696 trials of adjuvant high-dose interferon (HDI) with extended RFS compared with observations in approximately 2000 patients [3,29,30]. However, adverse events cannot be ignored. Thereinto, the E1684 trial reported 67.1% severe rates of toxicity as well as 7.0% laboratory toxicity, including severe myelosuppression, hematological toxicity, or renal dysfunction in the HDI arm [31]. Trial E1690 also reported 52.6% severe and 28.8% laboratory toxicity. By analyzing the quality of life in the AIM-HIGH study, Dixon et al. [32] found the worse health-related quality of life (HRQoL), symptom scores, and financial difficulties caused by even low-dose IFN. Mao et al. [33] reported that both the 4-week regimen and the 1-year regimen of HDI showed higher incidences of all-grade toxicities, especially hepatotoxicity and hematotoxicity. 

In former real-world clinical practice for advanced melanoma in China, most of the patients received a median or low dose of IFN-α due to poor tolerance of HDI, which may cause poor efficacy. In addition, China’s discontinued supply of imported IFN (mostly Interferon alfa-2b recombinant for injection) spurred us to investigate better alternatives to the adjuvant therapy of resected advanced-stage melanoma. As a matter of fact, a novel PD-1 inhibitor has been substituted for IFN and is widely used as an adjuvant setting in real-world practice in China. Li et al. [34] in the Guangzhou (CN) center showed that adjuvant anti-PD-1 treatment contributed to a significantly longer RFS (not reached vs. 7.8 mo) and DMFS (not reached vs. 9.4 mo; HR, 0.324; 95% CI, 0.122 to 0.861; *p* = 0.017) than HDI in Chinese patients with cutaneous melanoma, with the median follow-up times being 19.2 mo (PD-1) and 46.2 mo (HDI), respectively. However, no advantage was shown in RFS (HR 1.204; 95% CI, 0.521 to 2.781) and DMFS (HR 1.968; 95% CI, 0.744–5.209) in acral melanoma patients. Li et al. [35] in the Hangzhou (CN) center also reported no significant difference in RFS (not reached vs. 25 mo) for stage III melanoma between the pembrolizumab and IFN-α-2b groups (HR = 1.20, *p* = 0.75). In our report, the median RFS in the PD-1, IFN, and OBS groups was 23 months,15 months, and 11 months and the median DMFS was not reached, 72 months, and 20 months, respectively. A longer follow-up time may contribute to the difference when compared with other studies. In terms of subtype analysis, although the efficacy of the PD-1 inhibitor on acral melanoma patients was marginally significant (*p* = 0.05) as compared to the conventional therapy group (IFN+OBS), we still reached a non-statistically significant result, wehile the non-significance among the three groups in DMFS could be caused by the inherently poor outcome of PD-1 inhibitors in BRAF-mutant metastatic melanoma patients in real-world clinical practice [36].

Our study sheds new light on the effectiveness of PD-1 inhibitors, but it still has limitations. Firstly, considering the side effects brought by HDI, our study lacks a comparison between the standard HDI regimen and the PD-1 regimen, which was mentioned earlier. Secondly, there is still controversy about using PD-1 inhibitors for stage IIIA patients. Since all the stage IIIA patients enrolled in this study were in a positive sentinel lymph node status with a Breslow thickness over 1 mm, whether the overall IIIA population would benefit from existing adjuvant therapy remains inconclusive. Thirdly, even though this study included a considerable sample size, all our patients are Chinese within one single cancer center with a relatively short follow-up time; collaborations among international multi-center studies are needed to ensure a more applicable treatment regimen regarding the adjuvant therapy of high-risk resected melanoma, especially the acral subtype. Finally, the efficacy of adjuvant therapy with PD-1 mAb in Chinese-specific subtypes still needs to be improved at present. It is necessary to explore the combination regimen based on the anti-PD-1 inhibitor in prospective studies, or through neoadjuvant therapy to obtain better efficacy.

## 5. Conclusions

In conclusion, our study indicated that the adjuvant PD-1 inhibitor treatment was more effective than conventional therapy groups (IFN+OBS) in prolonging OS, RFS, and DMFS in Chinese patients with resected stage III acral and cutaneous melanoma, especially those with a heavy disease burden, stage IIIC/D, ulcerated, macrometastasis in the lymph nodes, or distant metastasis.

## Figures and Tables

**Table 1:** Demographics and clinicopathologic characteristics of the patients.

Characteristics	PD-1 (%) n = 126	IFN (%) n = 31	OBS (%) n = 42	*p* Value
Gender				0.201
Female	63 (50.0%)	11 (35.5%)	16 (38.1%)	
Male	63 (50.0%)	20 (64.5%)	26 (61.9%)	
Age				0.148
<60 yrs	60 (47.6%)	17 (54.8%)	14 (33.3%)	
≥60 yrs	66 (52.4%)	14 (45.2%)	28 (66.7%)	
Subtype				0.191
Acral	84 (66.7%)	18 (58.1%)	34 (81.0%)	
Cutaneous	34 (27.0%)	9 (29.0%)	7 (16.7%)	
Unknown primary	8 (6.3%)	4 (12.9%)	1 (2.4%)	
Gene Mutation				0.060
BRAF	23 (18.3%)	6 (19.4%)	12 (28.6%)	
KIT	10 (7.9%)	1 (3.2%)	4 (9.5%)	
NRAS	24 (19.0%)	2 (6.4%)	8 (19.0%)	
Wildtype	58 (46.0%)	13 (41.9%)	13 (31.0%)	
Untested	11 (8.7%)	9 (29.0%)	5 (11.9%)	
T stage				0.532
T0	8 (6.3%)	4 (12.9%)	1 (2.4%)	
T1	5 (4.0%)	3 (9.7%)	1 (2.4%)	
T2	16 (12.7%)	3 (9.7%)	5 (11.9%)	
T3	36 (28.6%)	10 (32.3%)	15 (35.7%)	
T4	61 (48.4%)	11 (35.5%)	20 (47.6%)	
Ulceration				0.573
No	40 (33.9%)	7 (25.9%)	11 (26.8%)	
Yes	78 (66.1%)	20 (74.1%)	30 (73.2%)	
Nodal Involvement				0.727
Micrometastasis	70 (55.6%)	15 (48.4%)	24 (57.1%)	
Macrometastasis	56 (44.4%)	16 (51.6%)	18 (42.9%)	
N stage				0.215
N1	56 (44.4%)	19 (61.3%)	18 (42.9%)	
N2	39 (31.0%)	9 (29.0%)	17 (40.5%)	
N3	31 (24.6%)	3 (9.7%)	7 (16.7%)	
Stage III Subgroup				0.217
IIIA	13 (10.3%)	4 (12.9%)	2 (4.8%)	
IIIB	15 (11.9%)	8 (25.8%)	9 (21.4%)	
IIIC	82 (65.1%)	18 (58.1%)	28 (66.7%)	
IIID	16 (12.7%)	1 (3.2%)	3 (7.1%)	

## Data Availability

Not applicable.

## Supplementary Materials

The following supporting information can be downloaded at: https://www.mdpi.com/article/10.3390/ph16010041/s1, Figure S1: Recurrence patterns for acral and cutaneous melanoma patients; Figure S2: Kaplan–Meier curves of RFS (A) and DMFS (B) for all enrolled acral and cutaneous patients stratified by adjuvant PD-1 inhibitor treatment versus conventional IFN treatment or observation; Figure S3: OS among all enrolled acral and cutaneous melanoma patients stratified by adjuvant PD-1 inhibitor treatment versus conventional IFN or observation, with a *p*-value of 0.019 between the PD-1 and the IFN groups; Figure S4: RFS for all stage IIIC and IIID melanoma patients stratified by adjuvant PD-1 inhibitor treatment versus conventional IFN or observation, with a *p*-value of 0.003 between the PD-1 and the IFN groups; Figure S5: RFS for all acral melanoma patients stratified by adjuvant PD-1 inhibitor treatment versus conventional IFN or observation, with a *p*-value of 0.075 between the PD-1 and the IFN groups; Figure S6: RFS for all enrolled patients with ulceration stratified by adjuvant PD-1 inhibitor treatment versus conventional IFN or observation, with a *p*-value of 0.056 between the PD-1 and the IFN groups; Figure S7: RFS for all melanoma patients with macrometastatic lymph nodes stratified by adjuvant PD-1 inhibitor treatment versus conventional IFN or observation, with a *p*-value of 0.075 between the PD-1 and the IFN groups; Table1: RFS of enrolled patients treated with the adjuvant PD-1 inhibitor versus conventional IFN or observation; Table S2: DMFS of enrolled patients treated with the adjuvant PD-1 inhibitor versus conventional IFN or observation.

## Abbreviations

AJCC: American Joint Committee on Cancer; CTCAE: Common Terminology Criteria for Adverse Events; CTLA-4: cytotoxic T lymphocyte-associated antigen-4; DMFS: distant metastasis-free survival; D+T: Dabrafenib+Trametinib; FUSCC: Fudan University Shanghai Cancer Center; HDI: high-dose interferon alpha-2b; HRQoL: health-related quality of life; ICI: immune checkpoint inhibitor; IFN: interferon; IFN-α2b: interferon-α-2b; ITM: in-transit metastases; PD-1: programmed cell death 1; PD-L1/2: PD ligand 1/2; pN: pathologic nodal; pT: pathological tumor; OBS: observation; OS: overall survival; RFS: relapse-free survival.
