# Supplementary material for: Adjuvant Anti-PD-1 Immunotherapy versus Conventional Therapy for Stage III Melanoma: A Real-World Retrospective Cohort Study
Source: Pharmaceuticals (Basel). 2022 Dec 28;16(1):41. doi: 10.3390/ph16010041 (PMC9867270; doi:10.3390/ph16010041)
Supplement: Supplementary file 1 [file pharmaceuticals-16-00041-s001.zip › pharmaceuticals-2097458-supplementary.pdf]

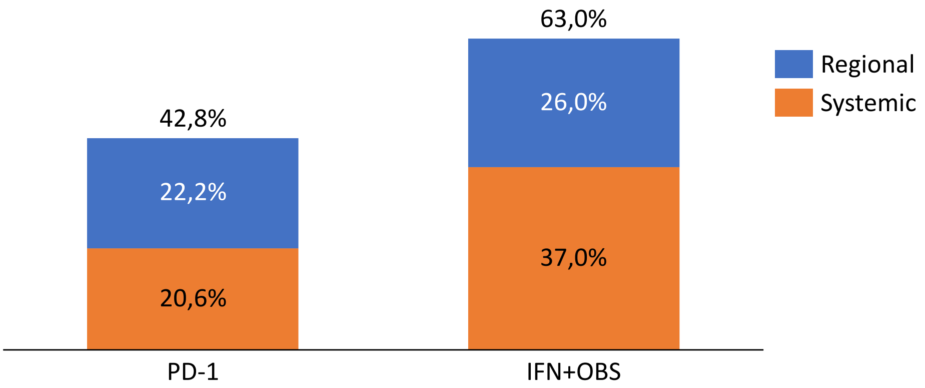

Figure S1. Recurrence patterns for acral and cutaneous melanoma patients.

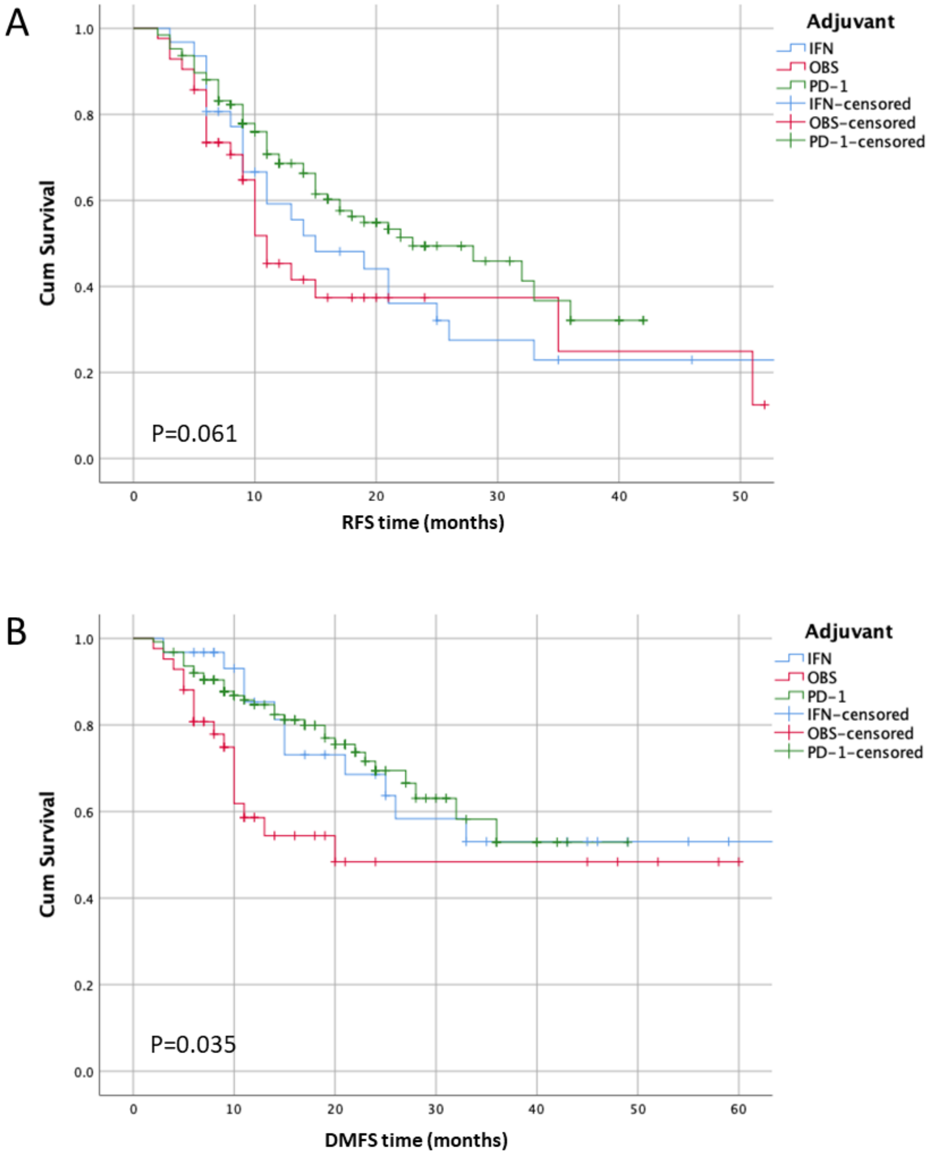

Figure S2. Kaplan-Meier curves of RFS (A) and DMFS (B) for all enrolled acral and cutaneous patients stratified by adjuvant PD-1 inhibitor treatment versus conventional IFN treatment or observation.

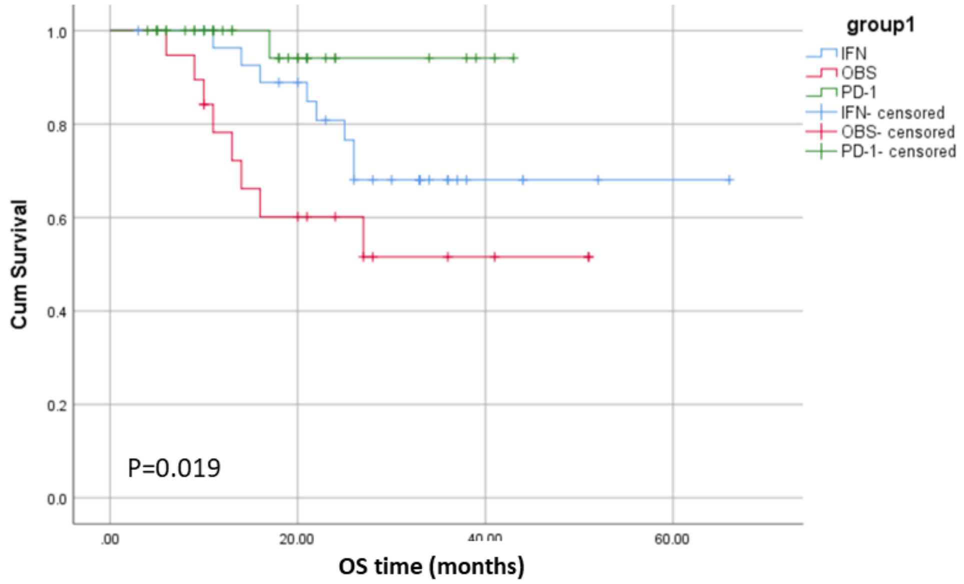

Figure S3. OS among all enrolled acral and cutaneous melanoma patients stratified by adjuvant PD-1 inhibitor treatment versus conventional IFN or observation, with a p-value of 0.019 between the PD-1 and the IFN groups.

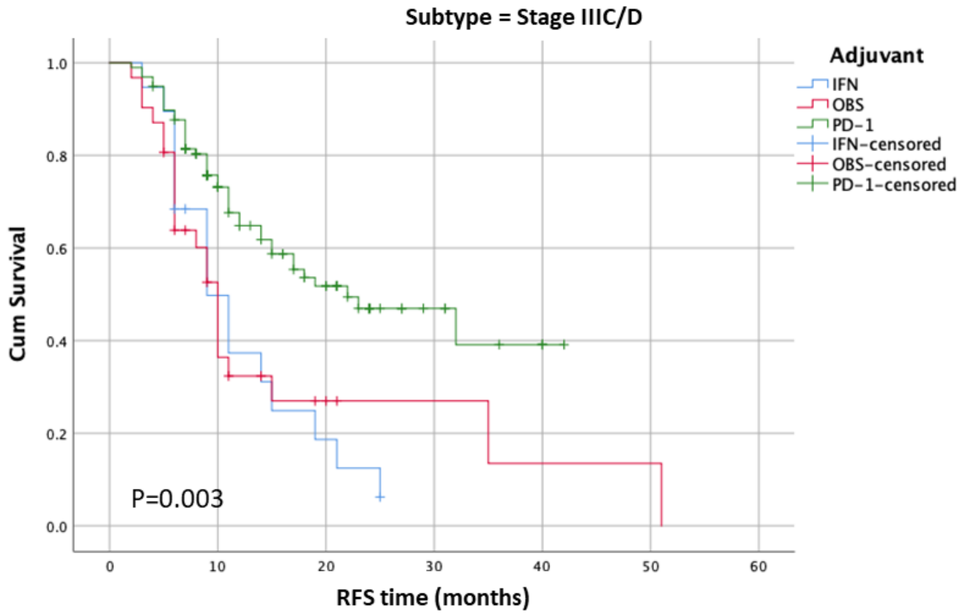

Figure S4. RFS for all stage IIIC and IIID melanoma patients stratified by adjuvant PD-1 inhibitor treatment versus conventional IFN or observation, with a p-value of 0.003 between the PD-1 and the IFN groups.

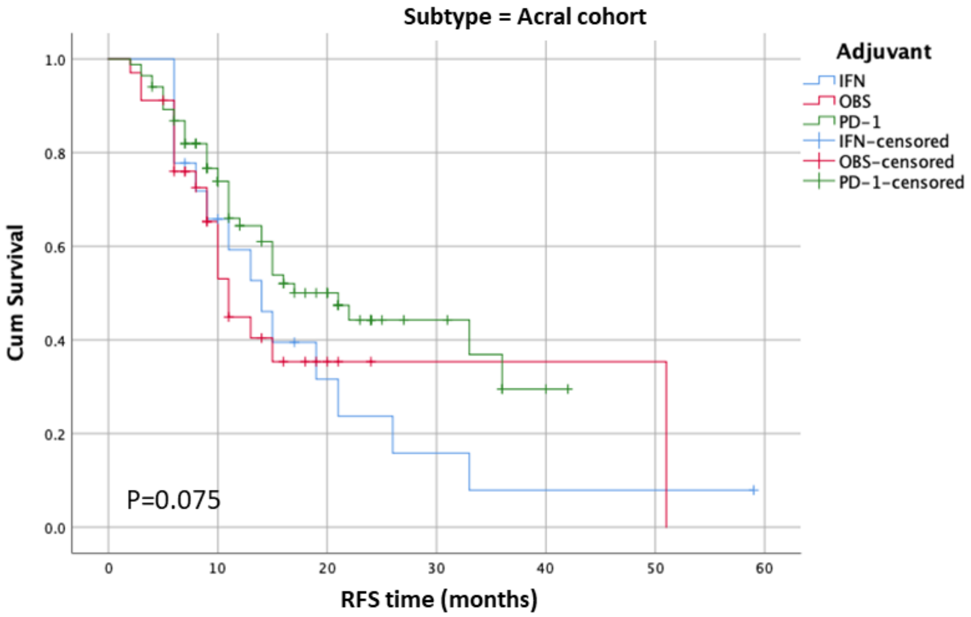

Figure S5. RFS for all acral melanoma patients stratified by adjuvant PD-1 inhibitor treatment versus conventional IFN or observation, with a p-value of 0.075 between the PD-1 and the IFN groups.

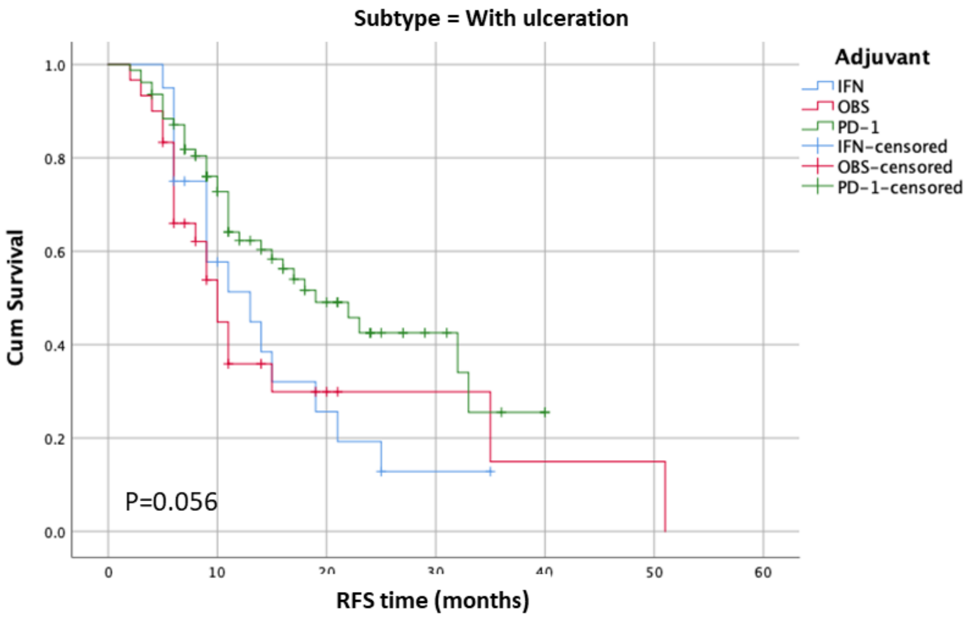

Figure S6. RFS for all enrolled patients with ulceration stratified by adjuvant PD-1 inhibitor treatment versus conventional IFN or observation, with a p-value of 0.056 between the PD-1 and the IFN groups.

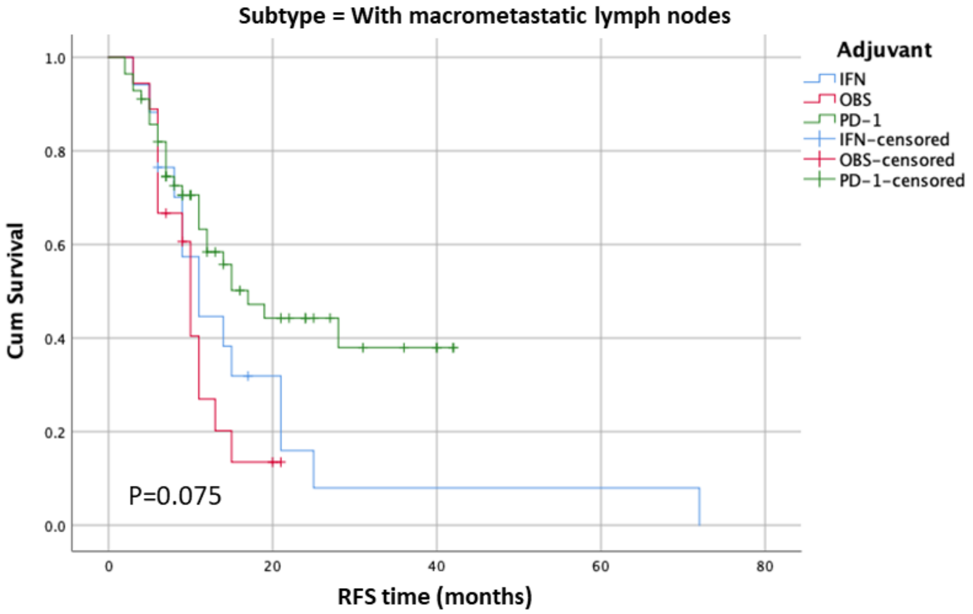

Figure S7. RFS for all melanoma patients with macrometastatic lymph nodes stratified by adjuvant PD-1 inhibitor treatment versus conventional IFN or observation, with a p-value of 0.075 between the PD-1 and the IFN groups.

Table S1. RFS of enrolled patients treated with the adjuvant PD-1 inhibitor versus conventional IFN or observation.

	mFP	mRFS	1-yr RFS	2-yr RFS	P value (vs PD-1)
PD-1	21	23	70.0%	49.4%	
IFN	24	15	59.2%	35.1%	0.170
OBS	49	11	45.3%	37.4%	**0.036**
IFN+OBS	46	13	51.8%	35.1%	**0.027**

Table S2. DMFS of enrolled patients treated with the adjuvant PD-1 inhibitor versus conventional IFN or observation.

	mFP	mDMFS	1-yr DMFS	2-yr DMFS	P value (vs PD-1)
PD-1	21	NR	84.7%	69.4%	
IFN	24	72	85.3%	68.5%	0.106
OBS	49	20	58.6%	48.4%	**0.014**
IFN+OBS	46	33	70.5%	57.3%	0.087
